# A Fast Method for Determination of Seven Bisphenols in Human Breast Milk Samples with the Use of HPLC-FLD

**DOI:** 10.3390/molecules28031432

**Published:** 2023-02-02

**Authors:** Szymon Szubartowski, Tomasz Tuzimski

**Affiliations:** 1 Department of Physical Chemistry, Medical University of Lublin, Chodźki 4a, 20-093 Lublin, Poland; 2 Doctoral School of Medical University of Lublin, Medical University of Lublin, Chodźki 7, 20-093 Lublin, Poland

**Keywords:** bisphenol (BPA), high-performance liquid chromatography (HPLC), dispersive liquid–liquid microextraction (DLLME), human breast milk samples, fluorescence detector (FLD), bisphenol A diglicydyl ether (BADGE), bisphenol F (BPF), endocrine disrupting chemical (EDC)

## Abstract

Plastic pollution, where bisphenol A (BPA) is widely used in its production, has gained popularity. BPA omnipresence and toxicity, especially for infants, has led food safety authorities to place restrictions on BPA usage. It has led to the introduction of the marked ‘BPA-free’-labelled products, where BPA is often replaced by other bisphenols (BPs) which are suspected of being similar or even more toxic than BPA. Moreover, the free forms of BPs are more dangerous than their conjugated forms and the conjugation of BPs is less effective in infants than in adults. Considering that human breast milk is the main source of nutrition for infants, the constant biomonitoring not only of BPA, but the wider group of BPs in such crucial matrices seems to be vital. In this study, a fast, simple, ‘green’ and cost-effective DLLME-based extraction technique combined with HPLC-FLD was optimized for the determination of seven selected bisphenols simultaneously. The procedure has satisfactory recovery values of 67–110% with the most RSD% at 17%. The LODs and LOQs ranged from 0.5 ng/mL to 2.1 ng/mL and 1.4 ng/mL to 6.3 ng/mL, respectively. The procedure was successfully applied to the biomonitoring of free forms of BPs in 10 real human breast milk samples.

## 1. Introduction

Products made of plastic are ubiquitous in everyday life. Plastics production has reached 6.3 billion tons, and it is predicted to quadruple in the future. They are usually an organic polymer with chemical additives which give plastic desired properties. The examples of compounds used as chemical additives are bisphenols (BPs). BPs can be leached out from plastic products such as plastic bottles or containers into beverages or food [1]. The most known compound from bisphenol group is bisphenol A. BPA is used as an additive to polycarbonate plastics, in the production of epoxy resigns or as a protective layer on the inside of the can protecting the metal against corrosion. BPA has been found in various body fluids such as urine, blood, breast milk, amniotic fluid or saliva and in tissues such as liver, brain or adipose tissue [2,3,4]. BPA has been suspected to have toxic effects such as causing endocrine disruption, heart disease, obesity, diabetes, hepatotoxicity, neurotoxicity, genotoxicity, mutagenicity, immunotoxicity, carcinogenicity or teratogenicity [5]. Therefore, BPA has been restricted in use [6] and completely banned in the manufacture of bottles intended for babies in the European Union (EU) [7]. Restriction in the use of BPA has affected the growing popularity of products labelled BPA-free where BPA is replaced with its analogue bisphenol S (BPS), bisphenol F (BPF) or bisphenol AF (BPAF) [8]. Unfortunately, BPA substitutes are not safer alternatives. Exposure to BPA analogues can lead to similar or greater toxic effects [8,9,10,11]. However, only a few BPA analogues such as BPS [12] or bisphenol A diglicydyl ether (BADGE) and its derivatives are currently under restrictions [13].

The main way that BPs come into the human body is by oral administration [14]. BPA is rapidly absorbed and conjugated in the liver into BPA-glucuronide and BPA-sulfate, and then excreted with the urine [14]. BPA analogues such as BPF or BPS exhibit a similar metabolic path. Conjugated forms have less endocrine effect, which make them safer than free forms. The most vulnerable group for free forms of BPs are fetuses and infants because their metabolism is less effective than in adults [14]. The main food for a newborn baby is breast milk which is perfect nutrition for infants containing not only sources of energy such as lipids, saccharides or proteins but also antibodies [15]. Apart from necessary and valuable components, human breast milk also contains xenobiotics harmful for infants such as BPs. BPA and tetrabromobisphenol A (TBBPA) have been found in the majority of 3021 human breast milk samples [16]. Therefore, human breast milk seems to be a critical body fluid to biomonitoring the level of BPs considering exposure group and the impact on their development.

Various techniques for the determination of bisphenols in human breast milk samples have been published so far. The most popular chromatographic technique for the determination of bisphenols in human breast milk samples is liquid chromatography coupled with tandem mass spectrometry (LC-MS/MS). LC-MS/MS offers low values of limits of detection (LOD) and quantification (LOQ) below 1 ng/mL, combined with a reliability of the obtained results [2]. One of the common sample preparation techniques used during biomonitoring of BPs in human breast milk samples is QuEChERS [4,17,18,19,20]. This technique contains the d-SPE (dispersive solid-phase extraction) step with the sample and can be appropriately cleaned up with dispersive salt or a mixture of salts [4], which is essential in the analysis of such complex matrices via LC-MS/MS. Solid-phase extraction (SPE) [21] and ultrasound assisted solid phase extraction (UA-SPE) [22] or d-SPE before SPE (d-SPE/SPE) [23] were also successfully performed during sample preparation.

An interesting alternative to previously presented sample preparation techniques can be dispersive liquid–liquid microextraction (DLLME) which was originally developed in 2006 for isolating poliaromatic hydrocarbons from water samples [24]. DLLME was successfully approached in the biomonitoring of BPs in human breast milk by F. Vela-Soria et al. [25] with the use of LC-MS/MS. The procedure was preceded by enzymatic hydrolysis with β-glucuronide which enables the total level of BPs to be determined.

In this study, the fast, cheap and ‘green’ DLLME sample preparation technique was optimized and quantitative analysis with the use of high-performance liquid chromatography coupled with fluorescence detector (HPLC-FLD) was performed as a cheaper alternative for LC-MS analysis obtaining satisfactory limits of quantification (LOQ) values. The optimized method was successfully used to determine seven BPs simultaneously in human breast milk samples from 10 women.

## 2. Results

### 2.1. Optimizing the Chromatographic Parameters

**Figure 1:**
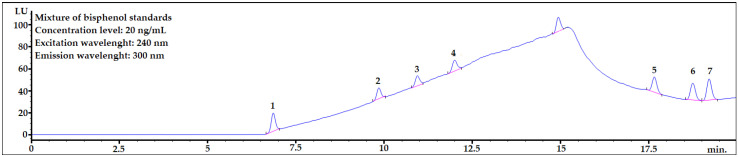
The mixture of bisphenol standards dissolved in ACN:H_2_O 1:1 (*v*/*v*). 1—BADGE∙2H_2_O, 2—BPF, 3—BPE, 4—BPA, 5—BADGE∙2HCl, 6—BADGE, 7—BPP.

**Figure 2:**
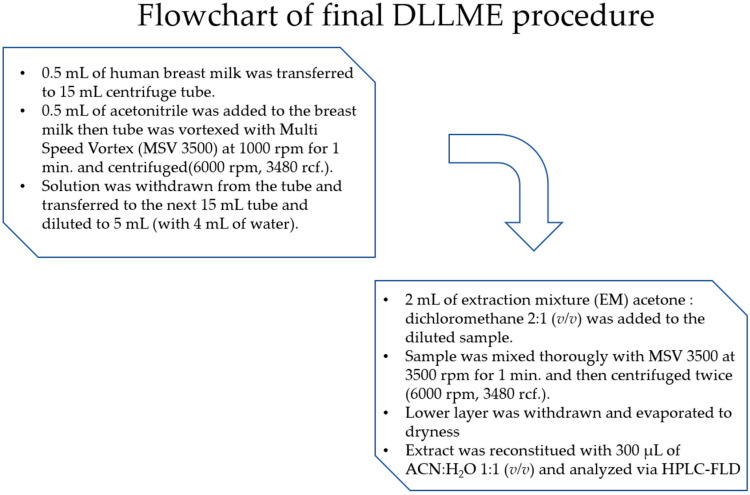
The flowchart of final DLLME procedure.

Chromatographic conditions were based on a previously published paper [3] with only slight changes. The details are shown in the Materials and Methods section. The exemplary chromatogram of the mixture of bisphenol standards in acetonitrile (ACN):water 1:1 (*v*/*v*) is shown in Figure 1. The mixture was prepared by dissolving 20 μL of MeOH solution of bisphenol standards in 980 μL of ACN:H_2_O 1:1 (*v*/*v*). The aim of this step was making solvent composition very similar to the final extract (please see Figure 2, last step of the final procedure) The concentration level was 20 ng/mL.

As seen in Figure 1, all analytes are well separated from each other. Baseline rising is caused by gradient elution. All analytes are eluted to 20 min. The peak at ~15 min. is BPAF which was removed from analysis in further studies. The symmetry factors for analytes were in the range 0.89–1.08.

### 2.2. Optimizing the Detection Conditions

**Figure 3:**
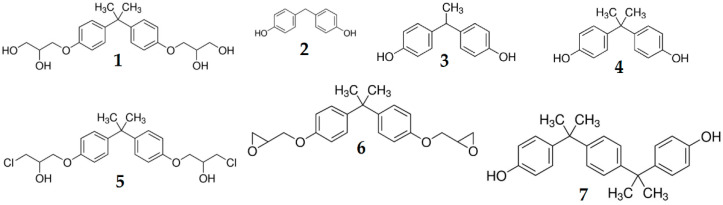
Chemical structures of all studied analytes. 1—BADGE∙2H_2_O, 2—BPF, 3—BPE, 4—BPA, 5—BADGE∙2Hl, 6—BADGE, 7—BPP.

Detection conditions were set based on fluorescent studies [26] for some of our analytes and our previous works [3,23,27]. For three analytes: BPF, BPE and BPA, the detector response is very similar. This is probably caused by very similar structures which differ only in the number of -CH_3_ groups linked to carbon connecting two phenol rings. For other analytes: BADGE∙2H_2_O, BADGE∙2HCl, BADGE and BPP, the detector response is stronger. These analytes contain more electronegative atoms, such as oxygen for BADGE and BADGE∙2H_2_O, chlorine for BADGE∙2HCl or an additional aromatic ring for BPP. The structures of all studied analytes are shown in Figure 3.

The excitation wavelength at 240 nm and emission wavelength at 300 nm with signal amplification set at 15 was found as the most optimal variation taking into account signal strength of analytes and the noise level.

The identification of bisphenols was based on comparing time retention in a mixture of bisphenol standards and in human breast milk samples.

### 2.3. Optimizing the DLLME Procedure for Human Breast Milk Samples

**Figure 4:**
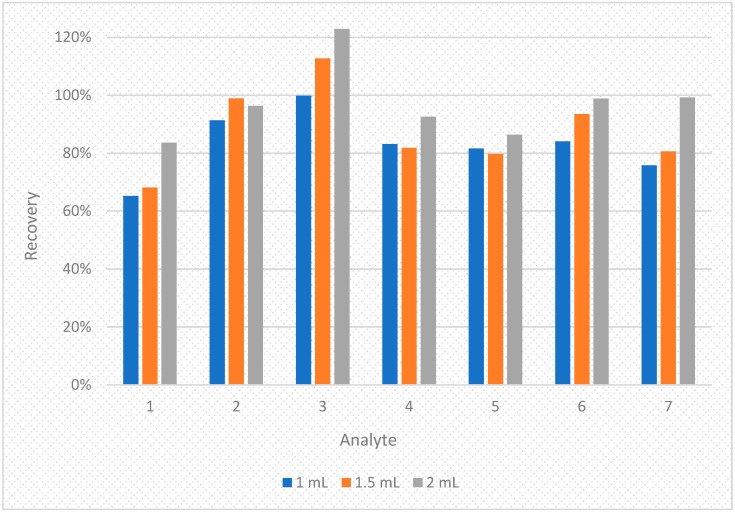
The diagram of comparison of the recoveries obtained by adding three different volumes of extraction mixture (1 mL, 1.5 mL and 2 mL) for seven bisphenols in spiked human breast milk samples. 1—BADGE∙2H_2_O, 2—BPF, 3—BPE, 4—BPA, 5—BADGE∙2HCl, 6—BADGE, 7—BPP.

The conditions for the preliminary studies were selected based on the literature [25,28,29,30,31,32] and authors’ own experience. The recovery values were 65.2–99.8%, 68.1–112.7% and 83.6–122.8% for 1 mL, 1.5 mL and 2 mL of the extracting mixture (acetone:dichloromethane 2:1 *v*/*v*) respectively. The results of this experiment are shown on diagram in Figure 4.

**Figure 5:**
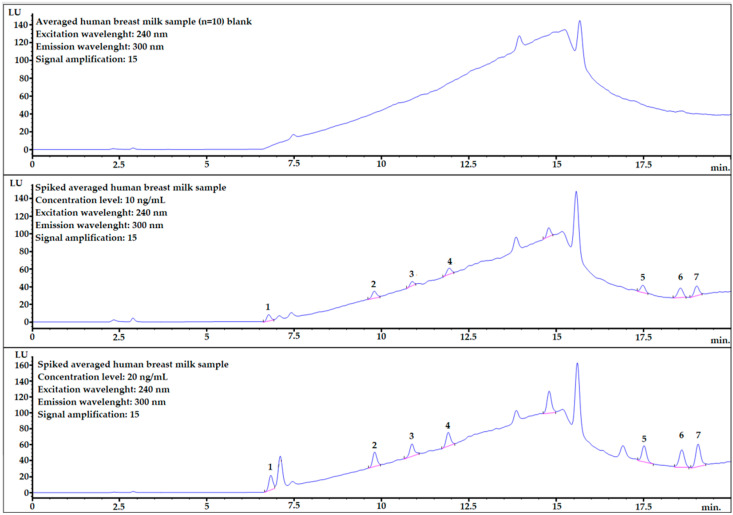
Example chromatograms of averaged human breast milk sample blank (**top**), and spiked samples at two different concentration levels: 10 ng/mL (**middle**) and 20 ng/mL (**bottom**). 1—BADGE∙2H_2_O, 2—BPF, 3—BPE, 4—BPA, 5—BADGE∙2HCl, 6—BADGE, 7—BPP.

As seen on the Figure 4, the best results were obtained for 2 mL of extracting mixture. Thus, this variant was chosen for further studies. Additional modifications such as adjusting the pH level, salting out or using ultrasound did not yield desired effects. Despite the complexity of human breast milk, the addition of d-SPE salts was not needed due to low matrix interferences (please see Figure 5 (top)).

The flowchart of final DLLME procedure is shown in Figure 2.

### 2.4. Recovery Studies

Recovery studies were performed for spiked samples at two different concentration levels: 10 ng/mL and 20 ng/mL in an averaged human breast milk sample. Choice was based on the highest LOQ values (please see Table 1) as the 10 and 20 ng/mL were approximately 2xLOQ and 4xLOQ, respectively.

The recovery values and RSD% for each analyte are presented in Table 2.

Recovery values range from 67% to 110% and RSD% ranges from 7% to 17% for all studied analytes. Previously, BPAF (t_R_~15 min.) was also analyzed but due to its low repeatability (RSD% > 25%), was removed from further analysis.

Most of the analytes are well separated from the matrix components. However, for analyte no. 1, the matrix component is very close (Figure 5 middle and bottom) but it seems to be fully separated considering that the baseline is starting to rise.

### 2.5. Quantitative Analysis

For reliable quantitative analysis, calibration curves were prepared in the averaged human breast milk sample blank and the concentration range was 0.5–25 ng/mL. The parameters obtained from the calibration curves are presented in Table 1. Formulas for calculation limits of detection (LODs) and limits of quantification (LOQs) are described in Section 4.

The values wee 0.5–2.1 ng/mL and 1.4–6.3 ng/mL for LOD and LOQ, respectively, which are the levels similar to those found in real various human samples [2]. Moreover, y-intercepts obtained from the calibration curves indicate the small influence of the matrix. R^2^ were above 0.99 for all the studied BPs.

The procedure was then applied for determination of BPs in 10 real human breast milk samples. BPA was the only bisphenol which was found and identified in 50% of the samples. The concentration did not exceed 4.7 ng/mL (LOQ value) in any sample. The results of quantitative analysis are presented in Table 3.

## 3. Discussion

The dominant detection technique in the biomonitoring of BPs in human samples is mass spectrometer coupled with LC or GC [2]. HPLC-FLD might be an effective and cheaper alternative for MS analysis. In this study, we managed to obtain LOD values below 1 ng/mL for four studied analytes (BADGE∙2H_2_O, BPE, BADGE∙2HCl, BADGE) which enables this technique to be used in the biomonitoring of BPs. The DLLME technique was successfully optimized with high recovery values and repeatability. The presented method can be used for the determination of only free forms of BPs. For the determination of conjugated and total level of BPs, an additional enzymatic hydrolysis step is required. The breast milk is a main source of food for infants. Therefore, the biomonitoring of free forms of bisphenols seems to be particularly important, considering less effective metabolism at the early stage of human life (please see Section 1). Compared to our previous study with the determination of the group of BPs in human breast milk samples via HPLC-FLD [23]:

- The sample preparation technique was significantly accelerated. Previously, the dispersive solid-phase extraction (d-SPE) before SPE (Oasis HLB column) was performed as a sample preparation technique. In this study, samples were prepared using the DLLME-based technique.
- The sample volume stayed at the same level (0.5 mL).
- The number of analyzed BPs stayed the same; however, in this study the most common bisphenol—BPA—was added to the study.
- The LOD and LOQ values were much lower than in previous wors. In this work, the LOD was 0.5–2.1 ng/mL, whereas in previous work, the LOD was 56.7–77.6 ng/mL.

**Figure 6:**
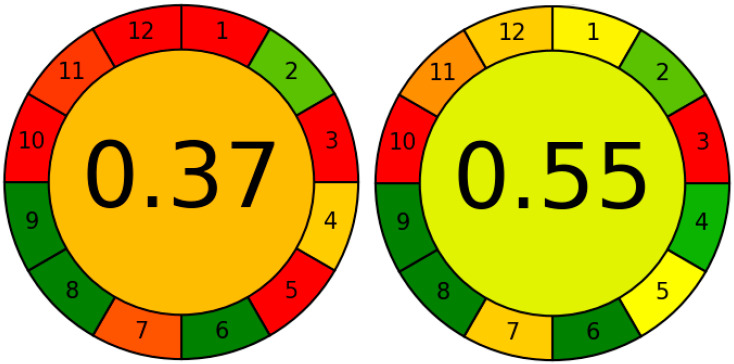
The comparison of two graphs comparing the greenness of two methods: (**left**) ([33] and (**right**) (this work) obtained from AGREE software. The meaning of each 12 points is presented in Section 4.9.

Apart from abovementioned improvements, the presented method is also more eco-friendly than previously published [23]. Figure 6 presents the comparison of scores obtained from the AGREE program.

The colour scale is explained in [33]. The dark red and dark green correspond to the values 0.0 and 1.0, respectively. The method from this work obtained a decent 0.55 score. Most of the principles are marked as green or yellow. Compared to our previous study, 6 out of 12 parameters were improved (1, 4, 5, 7, 11, 12), and the overall score has gone higher by 0.18. The improvement in 5, 11 and 12 (see Section 4.9) highlights the main advantages of the DLLME technique such as miniaturization, reducing the amount of harmful solvents which results in the safety for the operator. The two parameters marked as red are the location of the analytical device toward the investigated object (method is off-line–score 0.0) and reagents obtained from the renewable source (score 0.0). These two parameters are practically impossible to improve in the DLLME technique.

In sum, the presented DLLME-based sample preparation technique with HPLC-FLD analysis provides a reliable analysis of BPs in human breast milk samples. Lowering the usage of harmful solvents for both humans and the environment allows the method to be classified as ‘green’. Considering the low matrix effect, high recovery values and repeatability, the DLLME procedure might be preferred (if needed after slight modifications) to other chromatographic techniques such as LC-QqQ (triple quadrupole spectrometer) which is planned for the future.

## 4. Materials and Methods

### 4.1. Chemical Reagents and Bisphenols Standards

Methanol (MeOH), acetonitrile (ACN), dichloromethane (CH_2_Cl_2_) and acetone with hypergrade for liquid chromatography (purity ≥ 99.8%) were obtained from Sigma Aldrich (St. Louis, MO, USA). Formic acid (HCOOH) with purity ≥ 98% was obtained from Sigma Aldrich (St. Louis, MO, USA). Deionized water used in the sample preparation and as a mobile phase component was prepared in our laboratory using the Hydrolab System (Gdańsk, Poland).

All seven bisphenol standards (chemical structure are shown in Figure 3) with the purity ≥ 98% were purchased from Sigma Aldrich (Bellefonte, PA, USA): 3-[4-[2-[4-(2.3-Dihydroxypropoxy)phenyl]propan-2-yl]phenoxy]propane-1.2-diol (BADGE·2H_2_O), 4,4′-Methylenediphenol (bisphenol F—BPF), 1,1-Bis(4-hydroxyphenyl)ethane (bisphenol E—BPE), 4-[2-(4-hydroxyphenyl)propan-2-yl]phenol (bisphenol A–BPA), 1-Chloro-3-[4-[2-[4-(3-chloro-2-hydroxypropoxy)-phenyl]propan-2-yl]phenoxy]propan-2-ol (BADGE·2HCl), 2-[[4-[2-[4-(Oxiran-2-ylmethoxy)phenyl]propan-2yl]phenoxy]methyl]oxirane (BADGE) and 4,4′-(1,4-Phenylenediisopropylidene) bisphenol (BPP).

Bisphenols were dissolved with MeOH in glass flasks and mixtures with the desired concentration and composition were prepared by mixing the proper amount of each standard solution and diluting it with MeOH. BPs solutions were stored in the freezer in −23 °C.

### 4.2. Instrumental Analysis

The chromatographic equipment was the same and the gradient settings were set on the basis of previously the published method [3].

The chromatographic equipment consisted of a quaternary pump (Agilent 1200), an autosampler with a thermostat (Agilent 1260 Infinity II Vialsampler), a thermostat of column (Agilent 1200) and a fluorescence detector (Agilent 1260).

The separation was carried out on a Scherzo SM-C18 (150 mm × 4.6 mm) column with a 3 µm particle size (Agilent Technologies, Wilmington, DE, USA) thermostated at 22 °C. The mobile phase consisted of 50 mM formic acid (HCOOH) in water (component A) and 50 mM HCOOH in ACN. The gradient elution: 0–15 min from 40% to 75% component B; 15–15.5 min. from 75% to 85% component B; 15.5–20 min. isocratic elution 85% B. The flow rate was 0.45 mL/min. Samples were thermostated in an autosampler at 8 °C.

After every sample injection, the column was washed with 100% component B at 1.0 mL/min flow rate and then the column was conditioned with the isocratic elution with the initial composition (40% B) for 15 min.

### 4.3. Method Validation

Recovery studies, repeatability, selectivity, linearity, limits of detection (LOD) and quantification were performed with the use of spiked averaged human breast milk sample. An averaged human breast milk sample was formed by mixing milk from 10 different women. Samples were spiked with 20 μL of a mixture of bisphenol standards dissolved in MeOH at the beginning of the procedure directly to the human breast milk (see Figure 2). For blank samples, 20 μL of the mixture of bisphenol standards was swapped to 20 μL of pure MeOH. All laboratory equipment such as centrifuge tubes and automatic pipettes were checked for studied analytes’ contamination. All used solutions and mixtures were prepared and stored in freezer (−23 °C) in glass flasks.

### 4.4. Selectivity

Evaluation of selectivity was based on comparing the chromatograms from the averaged human breast milk sample blank, spiked human breast milk sample before the extracting procedure and spiked human breast milk after the procedure. The identification was based on comparing the retention times in the sample and in the mixture of bisphenol standards. Analysis was performed at 4 different excitation wavelengths: 225 nm, 230 nm, 235 nm and 240 nm, with the emission wavelength set at 300 nm. The wavelength selection criteria were described in detail in the Results section.

### 4.5. Linearity

To prepare the calibration curve, a few final averaged human breast milk sample blanks were connected and divided into equal parts, where the proper identical amount of mixture of bisphenol standards (5% of final volume) was added. A calibration curve was prepared based on the 8 concentration levels in the range of 0.5 ng/mL to 25 ng/mL. The signal amplification during linearity studies was set on 15. To evaluate linearity, coefficients of determination (R^2^) were analyzed.

The LOD and LOQ were obtained by formulas: 3.3 × (SD/S) and 10 × (SD/S), respectively, where SD is the standard deviation of the response (peak area) and S is the slope of the calibration curve. Analytes were identified by the basis of time retention. The signal amplification was set at 15. HPLC analysis was repeated three times.

### 4.6. Optimizing the DLLME Procedure

The optimizing procedure and recovery studies were performed on the averaged human breast milk sample, which was the mixture of equal amount of 10 breast milk samples from 10 different women. The extraction mixture (EM) composition was acetone:dichloromethane 2:1 (*v*/*v*). The EM was prepared in a glass flask by mixing proper volumes of solvents. Firstly, the volume of EM was checked (1 mL, 1.5 mL and 2 mL) for samples spiked at 50 ng/mL. Then, salting out, adjusting the pH level and the use of ultrasound bath were tried. Salting out was performed with the mixture of anhydrous MgSO_4_ and NaCl, such as in our previous work [4]. The amount of mixture of salts was downsized proportionally to sample size. The pH level was adjusted by addition of the formic acid or the ammonium. Moreover, the protein precipitation step was added at the beginning of the procedure by adding 0.5 mL of ACN directly to the human breast milk and centrifugation twice (6000 rpm., 3480 rcf.) and the precipitate was rejected for the further analysis. An amount of 1 mL (0.5 mL human breast milk + 0.5 mL ACN) was diluted to 5 mL with deionized water. EM was prepared right before the DLLME procedure.

The final optimized DLLME procedure is shown in Figure 2.

### 4.7. Recovery Studies

The recovery studies were performed on an averaged human breast milk sample, which was the mixture of equal amounts of 10 breast milk samples from 10 different women. In the preliminary studies, recoveries were evaluated in spiked samples at the concentration level of 50 ng/mL of sample for six replicates (*n* = 6). The final procedure was validated by repeating the DLLME procedure 18 times (*n* = 18) for 2 different concentration levels (10 ng/mL and 20 ng/mL), and six replicates per day (*n* = 6). For all seven analytes, relative standard deviation (RSD%) was calculated. The formulas used during recovery studies are shown below:

Recovery (%) = A/B × 100%

A—Peak area of the analyte obtained after procedure where sample were spiked before DLLME extraction.
B—Peak area of analyte obtained after procedure where sample were spiked after DLLME extraction directly into vial.

RSD% = C/D × 100%

C—Standard deviation of the recovery (%).
D—Mean recovery (%).

### 4.8. Sample Collection and Storage

Human breast milk samples were obtained from the Department of Obstetrics and Pathology of Pregnancy, Medical University of Lublin, Poland. Before, sampling breasts were cleaned with abundant water. Then, the 10–15 mL of 10 human breast milk samples were transferred into the centrifuge tubes and stored in the freezer at −23 °C. Samples were defrosted right before the procedure. 

This study was approved by the Bioethics Committee at the Medical University of Lublin, Poland (Resolution of the Bioethics Committee at the Medical University of Lublin No. KE-0254/239/2021).

### 4.9. Evaluation of ‘Greenness’ of the Presented Method

The evaluation was prepared with the Analytical GREEnness calculator (AGREE) prepared by Pena-Pereira et al. [33]. The open-source AGREE software was downloaded from https://mostwiedzy.pl/AGREE (accessed on 30 January 2023). The method was evaluated according to following 12 different principles marked as the number on the graph (see Figure 6): sample pretreatment activities (1), sample size (2), the location of the analytical device toward the investigated object (3), the number of steps (4), the level of automation and miniaturization of the sample preparation step (5), derivatization step (6), volume of analytical waste (7), the number of analytes determined in one hour (8), the use of energy (9), reagents obtained from renewable source (10), the total amount of toxic reagents (11), the safety of the operator (12). All 12 parameter weights were set at equal level—2 (possible range: 0–4). The software evaluated in the range of 0.0–1.0, where 1.0 is the greenest method.

## 5. Conclusions

To the best of our knowledge, this method is the first to combine the advantages of DLLME as extraction techniques with HPLC-FLD for the identification and quantification of bisphenols residues in human breast milk samples. Satisfactory recovery values, repeatability, and low LOQs of the presented procedure enable the biomonitoring of BPs in real human breast milk samples. BPs were determined in 10 human breast milk samples. The free forms of BPA have been detected in 50% of analyzed samples. BPA as the only analyte found in the samples may prove that it is the most commonly used bisphenol in the production, despite the growing popularity of products labelled ‘BPA-free’.

As mentioned in the introduction, free forms of bisphenol are more toxic than the conjugated and human breast milk is the main nutrition where BPs can enter the infant body. Moreover, the restriction described in the Introduction section and people’s awareness about toxicity of BPA might force producers to substitute BPA or bisphenol S (BPS), to BPs which are not under any restrictions in use. Therefore, developing new, ‘green’ and reliable methods for biomonitoring a group of BPs simultaneously is still needed. The biomonitoring of BPs in human breast milk samples will enable the estimation of the exposure of these xenobiotics to infants and might lead to extended restrictions on a wider group of these analytes. Thus, it will decrease the exposure to BPs for infants that provide many health problems in their later stages of life.

## Figures and Tables

**Table 1:** Method validation parameters obtained from calibration curves in averaged human breast milk sample (*n* = 10): time retention, linear regression, coefficient of determination, standard deviation (SD) of slope, limit of detection, limit of quantification.

No.	Bisphenol	t_R_ (min.)	Linear Regression	SD of Slope	Coefficient of Determination (R^2^)	LOD (ng/mL)	LOQ (ng/mL)
1	BADGE∙2H_2_O	~6.84	y = 14.87x + 0.60	0.30	0.9988	0.8	2.5
2	BPF	~9.84	y = 6.74x + 10.23	0.31	0.9940	2.1	6.3
3	BPE	~10.93	y = 9.91x + 4.47	0.16	0.9989	0.6	1.8
4	BPA	~11.98	y = 4.45x + 10.97	0.19	0.9929	1.5	4.7
5	BADGE∙2HCl	~17.64	y = 19.36x + 17.05	0.24	0.9994	0.5	1.4
6	BADGE	~18.73	y = 11.65x + 26.89	0.23	0.9985	0.7	2.2
7	BPP	~19.19	y = 11.735x + 12.38	0.62	0.9923	2.1	6.3

**Table 2:** The mean recovery values and relative standard deviations for all 7 studied analytes obtained during validation of the procedure (*n* = 18, *n* = 6 per day).

No.	Bisphenol	Concentration Level			
		10 (*n* = 18)		20 (*n* = 18)	
		Recovery (%)	RSD%	Recovery (%)	RSD%
1	BADGE∙2H_2_O	67%	11%	72%	11%
2	BPF	101%	11%	108%	16%
3	BPE	97%	10%	108%	17%
4	BPA	102%	16%	110%	8%
5	BADGE∙2HCl	88%	12%	90%	11%
6	BADGE	99%	11%	85%	7%
7	BPP	76%	7%	82%	11%

**Table 3:** Results of quantitative analysis for BPA in 10 human breast milk samples. ND—not detected, ~LOD—concentration level slightly above LOD, <LOQ—concentration level below LOQ.

Patient	Concentration Level
1	ND
2	<LOQ
3	~LOD
4	ND
5	ND
6	<LOQ
7	ND
8	ND
9	~LOD
10	~LOD

## Data Availability

Not applicable.

## Sample Availability

Samples of the compounds are not available from the authors.
